# Protective Functions of Reactive Astrocytes Following Central Nervous System Insult

**DOI:** 10.3389/fimmu.2020.573256

**Published:** 2020-09-30

**Authors:** Mathias Linnerbauer, Veit Rothhammer

**Affiliations:** ^1^ Department of Neurology, Klinikum rechts der Isar, Technical University of Munich, Munich, Germany; ^2^ Department of Neurology, University Hospital Erlangen, Friedrich–Alexander University Erlangen–Nürnberg, Erlangen, Germany

**Keywords:** protective, astrocytes, neuroinflammation, astrogliosis, neurodegeneration, multiple sclerosis, ischemic stroke, Alzheimer’s disease

## Abstract

Astrocytes play important roles in numerous central nervous system disorders including autoimmune inflammatory, hypoxic, and degenerative diseases such as Multiple Sclerosis, ischemic stroke, and Alzheimer’s disease. Depending on the spatial and temporal context, activated astrocytes may contribute to the pathogenesis, progression, and recovery of disease. Recent progress in the dissection of transcriptional responses to varying forms of central nervous system insult has shed light on the mechanisms that govern the complexity of reactive astrocyte functions. While a large body of research focuses on the pathogenic effects of reactive astrocytes, little is known about how they limit inflammation and contribute to tissue regeneration. However, these protective astrocyte pathways might be of relevance for the understanding of the underlying pathology in disease and may lead to novel targeted approaches to treat autoimmune inflammatory and degenerative disorders of the central nervous system. In this review article, we have revisited the emerging concept of protective astrocyte functions and discuss their role in the recovery from inflammatory and ischemic disease as well as their role in degenerative disorders. Focusing on soluble astrocyte derived mediators, we aggregate the existing knowledge on astrocyte functions in the maintenance of homeostasis as well as their reparative and tissue-protective function after acute lesions and in neurodegenerative disorders. Finally, we give an outlook of how these mediators may guide future therapeutic strategies to tackle yet untreatable disorders of the central nervous system.

## Introduction

**Figure 1:**
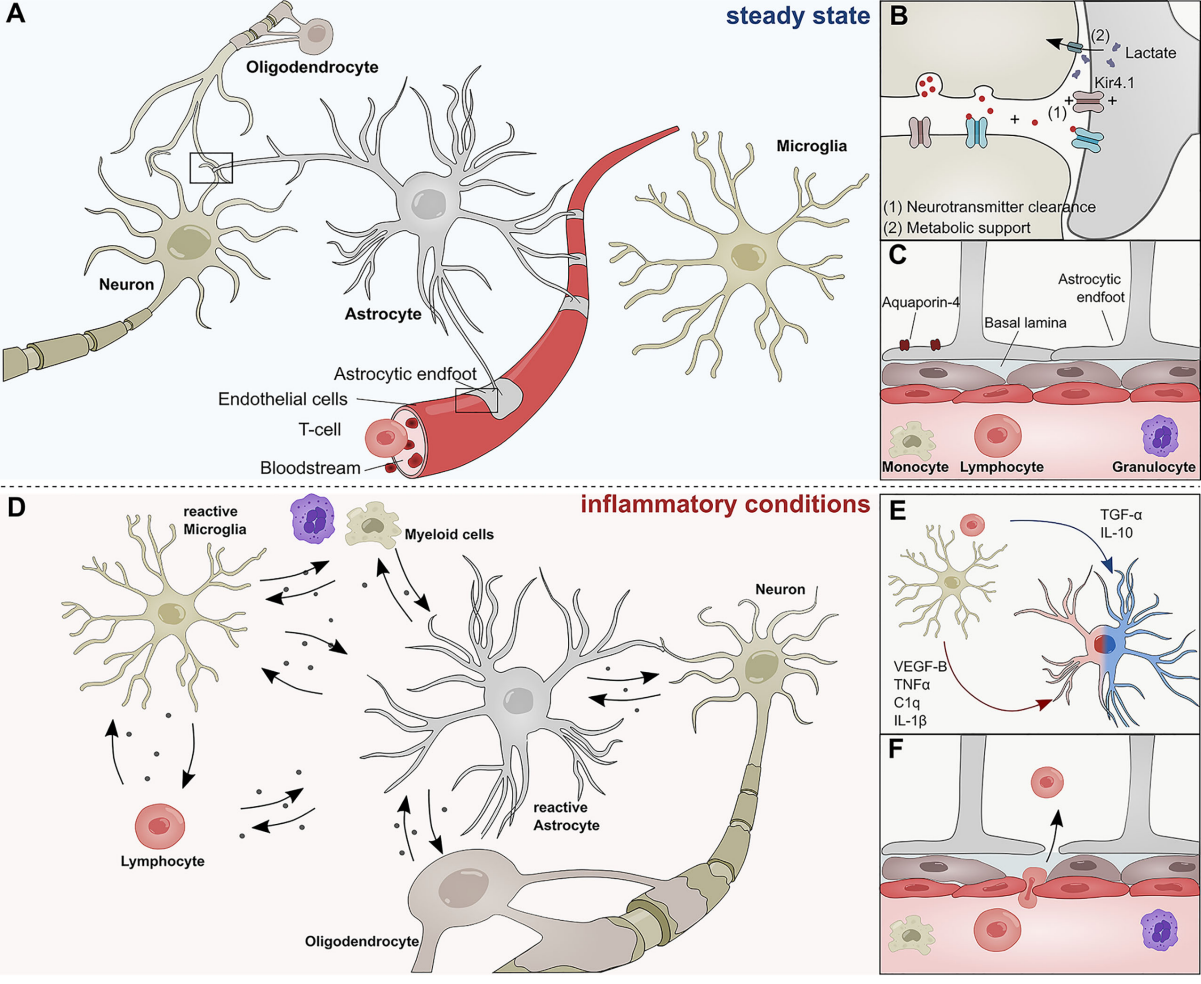
Role of astrocytes in the steady state and inflammatory conditions. **(A)** Astrocytes interact with neurons, oligodendrocytes, microglia, and cells of the BBB during steady state conditions. **(B)** Astrocytes form tripartite synapses with neurons and regulate their synaptic transmission through metabolic support and the clearance of neurotransmitters. **(C)** Astrocytic endfeet line the cerebral vasculature and are a constituent of the blood brain barrier, thus limiting the infiltration of pathogens and peripheral immune cells into the central nervous system. Their endfeet express high levels of Aqp-4 and form a close interaction with pericytes and the basal lamina of the brain parenchyma. **(D)** During inflammatory conditions, reactive astrocyte secrete a plethora of inflammatory mediators that regulate functions of myeloid cells, lymphocytes, oligodendrocytes, neurons, and microglia. **(E)** Soluble inflammatory mediators derived from mircoglia and other immune cells differentially induce pathogenic (red) or protective (blue) astrocyte functions. **(F)** Peripheral immune cells pervade the BBB during inflammatory conditions and transgress into the CNS. C1q, Complement component 1q; IL-1β, Interleukin-1 β; IL-10, Interleukin 10; TNF-α, Tumor necrosis factor α; TGF-α, Transforming growth factor α; VEGF-B, Vascular endothelial growth factor B.

Astrocytes are the most abundant cell type in the mammalian central nervous system (CNS) and responsible for a multitude of functions. During development, astrocytes arise from neural stem cells (NSCs) in the subventricular zone (SVZ) and migrate along radial glia processes to populate the CNS (1). Once their migration is complete, astrocytes differentiate into subgroups with a high degree of functional and regional specialization (1–6). During postnatal development, astrocytes instruct the formation of excitatory and inhibitory synapses, support developmental myelination, and aid the establishment of complex neural circuitry through the secretion of soluble factors (7–10) and physical cell contact (11–13). Throughout adulthood, astrocytes form close interactions with neurons to provide structural support and engage in metabolic coupling, serving as nutrient source and storage for neurons (**Figures 1A, B** **)** (14). Particularly lactate produced by astrocytes has been demonstrated to play an important role in the modulation of neuronal excitability and plasticity (15). Furthermore, astrocytes actively take part in synaptic transmission and have been shown to modulate cognitive functions through the clearance of neurotransmitter and other extracellular factors (16–20) (**Figure 1B**). In this context, the inward rectifying K^+^ channel Kir4.1 has gained attention as part of a K^+^ spatial buffering system that is required for neuronal transmission and functioning (**Figure 1B**). Kir4.1 is highly expressed in astrocytic endfeet, and its misregulation has been linked to numerous neurological disorders (21–23).

Besides their versatile role during neurogenesis and their contribution to the maintenance of neuronal circuitry, astrocytes are key participants in the formation and maintenance of the blood brain barrier (BBB) (24, 25) (**Figure 1C**). During CNS angiogenesis, astrocytes extend their polarized endfeet around the abluminal side of cerebral blood vessels and aid early sprout guidance and maturation by the expression of transporters, anti-permeability proteins and the secretion of growth factors (24–26). As key constituent of the glial limitans, astrocytic endfeet line the basement membrane surrounding the cerebral vasculature and provide a physical barrier between CNS and the peripheral blood system, thus limiting the influx of pathogens and large hydrophilic molecules (**Figure 1C**) (24, 25, 27–29). Furthermore, astrocytes control water homeostasis in the CNS *via* Aquaporin-4 (Aqp4) and other channel proteins involved in bidirectional fluid exchange across the BBB (30) (**Figure 1B**). The importance of Aqp4 in the CNS is demonstrated in a series of publications that link the (mal-)function of Aqp4 to multiple neurological disorders (31–34). Aqp4 has also been identified as a major target of autoantibodies in patients suffering from neuromyelitis optica (NMO), a rare CNS inflammatory disorder that has historically been closely associated to MS (35).

In addition to their versatile functions in the steady-state, astrocytes sense and react to danger signals in a multistep process referred to as astrogliosis (36, 37). Combinatorial exposure to a broad spectrum of extracellular cues, including cytokines, growth factors, and hormones induces transcriptional remodeling, resulting in cellular hypertrophy, proliferation and secretion of inflammatory mediators (**Figure 1D**) (36). The severity and permanence of these transcriptional changes is dependent on the type and strength of the stimuli and can range from reversible alterations to severe astrogliosis with compact scar formation (36, 38). Most forms of astrogliosis share the upregulation of glial fibrillary acidic protein (GFAP), a phenomenon that has been observed in multiple CNS disorders (1, 39–41).

For many decades, it was believed that severe astrogliosis and the formation of a glial scar inhibits axonal re-growth and is detrimental for neurological outcome. However, an increasing amount of evidence suggests that astrocytes also play beneficial roles in disease (42, 43). Methodological advances in the genomic analysis of reactive astrocytes have begun to shed light on the molecular mechanisms that define the fine line between pathogenic and protective astrocyte functions. For instance, a landmark study by Zamanian and colleagues (44) demonstrated that astrocytes respond differentially to varying forms of CNS insult. While exposure to lipopolysaccharide (LPS) resulted in the upregulation of pro-inflammatory genes and skewed astrocytes toward a cytotoxic profile, ischemia induced transcriptional programs that are associated with neuroprotective functions (44–47). In this context, particularly intercellular crosstalk with microglia has been identified as key regulator of astrocyte functions. Work by several groups including ours has unraveled molecular mechanisms through which microglia-derived molecules such as interleukin (IL)-1β, IL-10, tumor necrosis factor (TNF)-α, vascular endothelial growth factor (VEGF)-B, or transforming growth factor (TGF)- α, among others, modulate transcriptional programs in astrocytes that are associated to degenerative or protective functions (**Figure 1E**) (45, 48). In addition to microglia, numerous other CNS-resident and non-CNS-resident cell types modulate astroglial properties and are themselves subject to factors secreted by reactive astrocytes under inflammatory conditions (49). For instance, reactive astrocytes use contact- and diffusion-mediated mechanisms to modulate trafficking of peripheral immune cells into the CNS, a topic that has been extensively reviewed by Sofroniew and others (25, 28) (**Figure 1F**). Once the peripheral cells have extravasated, they accumulate in perivascular spaces where they are in close contact to astrocytic endfeet (50). It is possible that during this stage, MHCII^+^ astrocytes function as antigen-presenting cells to reactivate infiltrating lymphocytes and promote inflammation (51–53). Furthermore, there is increasing evidence that astrocytes control the survival of T-cells and B-cells *via* co-regulatory and secreted factors. Indeed, while FasL expression by astrocytes induces cell death in infiltrating lymphocytes, B cell–activating factor of the tumor necrosis factor (TNF) family (BAFF) produced by astrocytes promotes B-cell survival in inflammatory conditions and primary B cell lymphoma (54–56). Interestingly, astrocytes themselves respond to myeloid-derived APRIL, another member of the TNF superfamily with an increase in IL-10 production, consequently suppressing pro-inflammatory T-cell functions (57). These interactions between reactive astrocytes and cells of the adaptive immune system are complemented by their functions as part of the cerebral innate immune system (58).

Another degree of complexity is added when analyzing the temporal dynamics of astrogliosis in the context of disease. *In vivo* ablation experiments of astrocytes in experimental autoimmune encephalomyelitis (EAE), an animal model of Multiple Sclerosis (MS), demonstrated that astrocytes are required for disease suppression in early EAE stages, as loss of astrocytes worsened disease, characterized by increased BBB permeability, leukocyte infiltration, and neuronal death (59–62). Conversely, selective ablation of reactive astrocytes during the chronic phase of EAE ameliorated disease, marked by decreased microglial activation and monocyte infiltration (60). This and other studies underline the dire need to further dissect the contribution of astrocytes to the pathogenesis and progression of numerous CNS disorders.

While many studies focus on the pathogenic potential of reactive astrocytes, molecular mechanisms underlying their protective effects remain elusive at large. Here, we will discuss astrocyte-derived mediators with anti-inflammatory or tissue-protective properties, and examine how these factors may guide future therapeutic strategies. In this context we will not focus on protective astrocyte functions mediated by inflammatory cytokines or cell-cell contact, which have been reviewed extensively elsewhere (49, 63, 64), but rather concentrate on soluble factors often overlooked in the field of neuroinflammation.

## Protective Effects of Reactive Astrocytes Following CNS Insult

A widely recognized protective function of astrogliosis is the formation of a physical barrier, which limits the influx of peripheral immune cells and thus restricts lesion size (28, 65–67). This function has been discussed in depth in a series of excellent reviews (24, 25, 28, 37, 63). Here, we will focus on astrocyte secreted mediators relevant for astrocyte protective functions. Advances in single cell sequencing, spatial transcriptomics, and conditional knock-down approaches demonstrate that reactive astrocytes secrete a plethora of anti-inflammatory and tissue-protective mediators that act on numerous cells to control their inflammatory state (**Table 1**). This review will focus on three major domains to summarize the existing knowledge on astrocyte protective function: neurotrophic factors, neuropoetic cytokines, and growth factors.

**Table 1:** Tissue-protective mediators secreted by astrocytes.

Mediator	Disease model	Protective effect	References
BDNF	Ischemia; SCI; EAE	Promotes neuronal survival; increases remyelination	(68–70)
NGF	SCI; TBI; EAE	Pro-NGF induces neuronal death; mature-NGF promotes T_H_2 differentiation, neuronal survival and increases phagocytosis of microglia	(71–74)
GDNF	PD; EAE	Promotes neuronal survival; increases tight junction function; regulates microglial activation	(75–80)
CNTF	EAE; SCI;	Increases neuronal survival, promotes tight junction functions; increases remyelination	(81–83)
MANF/CDNF	Ischemia; AD; ER stress	Reduces pro-inflammatory cytokine production; promotes neuronal survival	(84–86)
PDGF family members	Acute and chronic demyelination	Increases OPC population density; regulates oligodendrocyte differentiation and proliferation	(87, 88)
FGF family members	Ischemia; SCI; viral induced demyelination	Promotes neuronal survival; regulates oligodendrocyte differentiation and proliferation; reduces glial reactivity	(89–91)
HB-EGF	*in vitro*	Increases neuronal survival	(92, 93)
IGF	TBI	Promotes neuronal survival	(94–96)
TGF-β	Ischemia; Toxoplasma infection	Reduces myeloid cell activation and pro-inflammatory cytokine production; promotes neuronal survival	(97, 98)
LIF	EAE; SCI; TBI	Increases stem cell renewal, promotes oligodendrocyte differentiation and myelination	(99–108)

### Neurotrophic Factors

Neurotrophic factors (NTFs) play an essential role in the growth, differentiation, and survival of neurons in health and disease. They can broadly be divided into neurotrophins, members of the ciliary neurotrophic factor (CNTF) family, and members of the glia derived neurotrophic factor (GDNF) family. While their role in the survival of neurons is relatively well defined, little is known about inflammatory functions and how astrocytes contribute to their production. Generally, glial cells are known to express low levels of NTFs under homeostatic conditions, but significantly upregulate their production following CNS damage (109, 110).

#### BDNF

**Figure 2:**
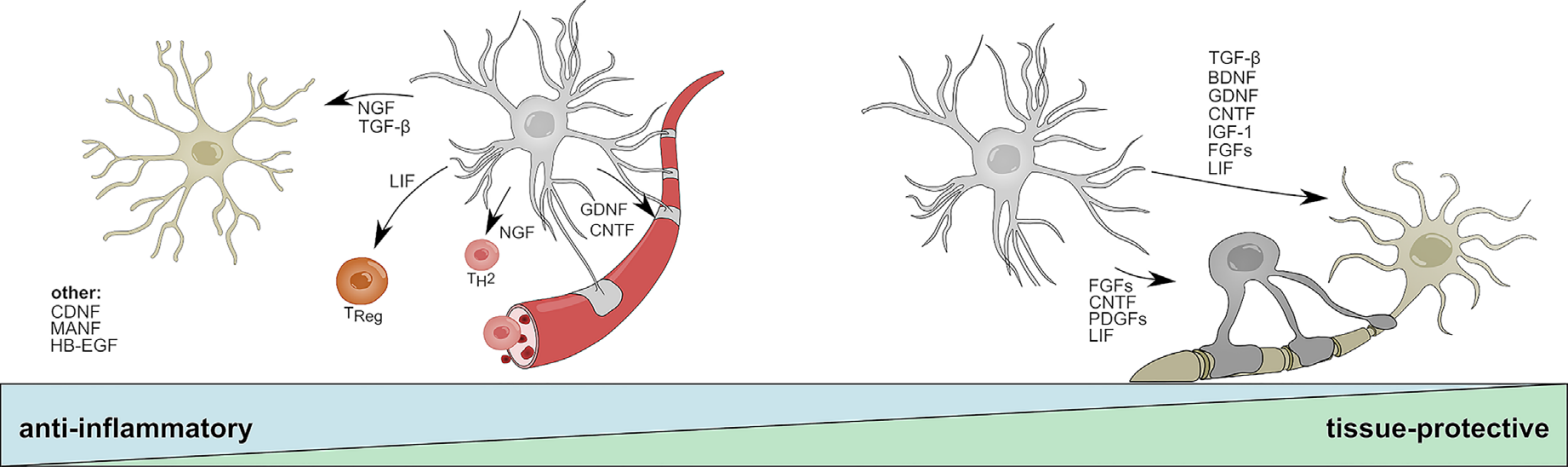
Anti-inflammatory and tissue-protective functions of reactive astrocytes. Activated astrocytes secrete soluble mediators with anti-inflammatory functions that help to resolve acute inflammation following CNS insult. NGF and TGF-β promote beneficial functions in microglia; LIF skews CD4 T-cells towards a regulatory phenotype; NGF promotes the differentiation into T_H_2 cells; GDNF and CNDF have beneficial effects on blood brain barrier permeability. CNDF, MANF, and HB-EGF have been associated to anti-inflammatory functions on multiple cell types or cells that are not displayed. During later stages, astrocyte-derived mediators promote the survival of neurons and oligodendrocytes and aid the long-term regeneration following CNS insult. TGF-β, BDNF, FGF family members, DNF, CNTF, IGF-1, and LIF increase neuronal survival; CNTF, LIF, and PDGF family members promote oligodendrocyte differentiation and myelination. BDNF, Brain derived neurotrophic factor; CNTF, Ciliary neurotrophic factor; FGF, Fibroblast growth factor; GDNF, Glial cell line-derived neurotrophic factor; HB-EGF, Heparin-binding epidermal growth factor; IGF-1, Insulin-like growth factor 1; LIF, Leukemia inhibitory factor; MANF, Mesencephalic astrocyte-derived neurotrophic factor; NGF, Nerve growth factor; PDGF, Platelet-derived growth factor; T_H_2, T helper type 2 cell; T_Reg_, T regulatory cell; TGF-β, Transforming growth factor β.

Brain-derived neurotrophic factor (BDNF) and nerve growth factor (NGF) are members of the neurotrophin family and highly expressed by astrocytes during development (109, 111–113). Throughout adulthood, astrocytes express low levels of BDNF but significantly upregulate its production in response to spinal cord injury (SCI) (114, 115), ischemia (115), and neuroinflammation (68, 69). BDNF signals through two receptors, the high-affinity TrkB receptor and the low-affinity p75^NTR^ receptor, both of which are expressed throughout the CNS by neurons, astrocytes, and oligodendrocytes (116). While BDNF/TrkB signaling on neurons has been shown to promote survival and neurite outgrowth (117), p75^NTR^ signaling induces apoptosis in cultured neurons (118). This dualistic signaling system corresponds to the dichotomic effector functions of BDNF. Early studies in the context of axotomy and SCI demonstrated beneficial effects of BDNF on the regeneration and long-term survival of neurons (119, 120) **(** **Figure 2** **)**. In EAE, reports suggest that BDNF depletion in CNS resident cells during the initial phase worsens disease, while deletion during later stages does not lead to significant differences (121). Although this protective effect might depend on multiple cell types, astrocytes have been suggested to be a key participant in BDNF-dependent remyelination in the cuprizone model of de- and remyelination (68). This is supported by observations of increased progenitor cell proliferation and maturation of neurons following lentiviral overexpression of *Bdnf* in hippocampal astrocytes (70) **(** **Figure 2** **)**. Furthermore, a study by Linker and colleagues (69) demonstrated that conditional depletion of BDNF in astrocytes worsens EAE severity. Interestingly, the authors did not observe changes in infiltrating immune cells, but demonstrated a significant increase in axonal loss and demyelination.

Overall, these findings suggest that BDNF regulates axonal myelination and neuronal function through Trk/p75^NTR^ signaling on neurons and potentially oligodendrocytes, making it a key constituent of neuronal health. This also becomes clear in the context of multiple neurodegenerative disorders, where a single nucleotide polymorphism (SNP) in the BDNF gene is associated to increased susceptibility, incidence and severity of MS (122–124) and Alzheimer’s disease (AD) (125), correlating with cognitive dysfunction (126, 127). Although the relative contribution of BDNF producing cells to the protective effects of BDNF remains under debate, a substantial body of evidence points to astrocytes as key drivers of BDNF mediated effects in disease. Furthermore, astrocyte-derived BDNF has been identified as mediator of the therapeutic functions of glatiramer acetate (GA), a FDA-approved drug for the treatment of relapse-remitting MS (RRMS) in a mouse model of neurodegeneration, demonstrating the potential of astrocyte-derived BDNF for future therapeutic strategies (128). Nonetheless, recent evidence suggest that there is a fine line between protective and pathogenic astrocyte-mediated functions of BDNF, as astrocytes themselves respond to increased levels of BDNF with the secretion of neurotoxic amounts of nitric oxide (NO), demonstrating a sophisticated feedback loop that prevents excessive BDNF signaling (129).

#### NGF

In contrast to BDNF, little is known about the immunomodulatory and tissue-protective functions of astrocyte-derived NGF. Early studies suggest upregulation of *Ngf* mRNA in astrocytes in models of traumatic injury, Parkinson’s disease (PD), and neuroinflammation (130–132). Similar to BDNF, mature NGF is cleaved from its precursor pro-NGF and signals *via* a dual receptor system consisting of TrkA and p75^NTR^ (133). While mature NGF preferentially binds to TrkA and promotes neuronal survival, pro-NGF has a higher affinity to p75^NTR^ and has been shown to induce apoptotic signaling in oligodendrocytes and neurons (134, 135). Under homeostatic conditions, pro-NGF and its mature form signal synergistically through TrkA/p75^NTR^ to promote the survival and differentiation of neuronal cells; however, imbalances in the relative abundances of TrkA and ^p75NTR^ have been described in multiple CNS disorders (133, 136–138). Interestingly, several reports demonstrate that activated astrocytes secrete increased amounts of neurotoxic pro-NGF *in vitro* and following SCI, suggesting a tissue-destructive role of endogenous, astrocyte-derived pro-NGF (71, 72). In contrast, treatment with exogenous NGF has been shown to be beneficial in models of traumatic injury and neuroinflammation (73, 74). For instance, administration of human NGF into the ventricle of marmoset monkeys prevented the development of lesions in an EAE model by skewing infiltrating T-cells towards an anti-inflammatory T_H_2 phenotype (73) **(** **Figure 2** **)**. In line with this observation, a recent study reports that NGF instructs TrkA-mediated phagocytosis of neurotoxic Amyloid-β plaques by microglia in a mouse model of AD (74) **(** **Figure 2** **)**. While it remains to be seen, which impact astrocyte-derived NGF has on the net effect of NGF, the activation of astrocytes and subsequent induction of *Ngf* expression by inflammation or stress-related events may contribute to both beneficial and harmful effects of NGF, depending on the availability of pro-NGF *vs.* mature NGF and the presence of TrkA vs. p75^NTR^ receptors on adjacent cells (72, 139). Of note, in addition to effects mediated by astrocyte derived NGF, a suppressive function of exogenously administered NGF in reactive astrocytes has been proposed, implicating a potential autocrine feedback loop that limits excessive astroglial activation (140).

#### GDNF

The GDNF family of neurotrophic factors consists of GDNF, neurturin (NTRN), artemin (ARTN), and persepin (PSPN) (141). All four members belong to the TGF-β superfamily and signal through the RET Tyrosine kinase to regulate the differentiation and survival of multiple distinct sets of neurons (141, 142). Interestingly, alternative signaling receptors, such as the neural cell adhesion molecule (NCAM) have been proposed and numerous studies suggest synergistic signaling with NGF, BDNF, and TGF-β (143–147). Reactive astrocytes rapidly upregulate the production of GDNF in response to LPS, IL-1β, IFN-γ and microglia-derived TNF-α, and have been shown to promote the survival of dopaminergic neurons *in vitro* (75, 76). This is in line with *in vivo* studies that demonstrate beneficial effects of astrocyte-specific overexpression of *Gdnf* in models of Parkinson’s disease (PD) (77, 78). Furthermore, transplantation of human NPCs committed to a glial fate that have been genetically engineered to overexpress GDNF promoted neuronal survival and regeneration in primate models of amyotrophic lateral sclerosis (ALS) (148, 149) **(** **Figure 2** **)**. Besides the supportive effects of astrocyte-derived GDNF on neurons, GDNF/GDNFRα signaling has been shown to promote the trans-endothelial resistance in an *in vitro* BBB model, suggesting a positive effect of astrocyte-derived GDNF on tight-junction function and BBB permeability during neuroinflammation (79) **(** **Figure 2** **)**. Collectively, further investigation into the anti-inflammatory and tissue-protective effects of astrocyte-derived GDNF is needed, but given the synergistic signaling of GDNF in combination with TGF-β and other NTFs, astrocytic GDNF may contribute to the reduction of inflammation and regenerative capacities following CNS insult.

#### CNTF

CNTF composes a separate family of neurotrophic factors and has been extensively studied as inducer of neuronal differentiation, survival and neurite outgrowth (150). Besides its effect on neurons, CNTF has been shown to support the maturation of oligodendrocytes and astrocytes (151–154) **(** **Figure 2** **)**. It signals through a heterotrimeric receptor complex consisting of the non-signaling subunit ciliary neurotrophic factor receptor alpha (CNTFRα), and the two signaling chains glycoprotein-130 (gp130) and leukemia inhibitor factor receptor (LIFRβ), which are shared with the distantly related leukemia inhibitory factor (LIF) and interleukin-6 (IL-6) (155). Upon CNTF binding, heterodimerization of gp130 and LIFRβ induces JAK/STAT dependent transcriptional programs that are associated with the differentiation and survival of neurons (156). Under homeostatic conditions, the expression of low levels of *Cntf* in astrocytes is limited to the white matter, indicating region-specific effects on distinct neuronal subpopulations (157). Interestingly, this finding is concordant with observations of increased *Cntf* expression in astrocytes and the upregulation of CNTFRα on neurons located in white matter lesions of MS patients (158). A study investigating the spatial and temporal dynamics of multiple NTFs in a cuprizone model of demyelination suggests that astrocytes express CNTF in a biphasic manner during initial demyelination and remyelination (159). Mechanistically, it has been proposed that loss of physical interaction between astrocytes and neurons following injury induces STAT3-mediated *Cntf* expression in astrocytes, which promotes survival of neurons and oligodendrocytes and may counteract TNF-α induced myelin disintegration during EAE (81–83) **(** **Figure 2** **)**. Similar to GDNF, beneficial effects on BBB permeability and a reduction of immune cell infiltrates have been observed following administration of exogenous CNTF in a mouse model of neuroinflammation (160) **(** **Figure 2** **)**. Collectively, the current data indicates that astrocyte derived CNTF might contribute to the reduction of acute inflammation and increases the survival of neurons and oligodendrocytes in the context of CNS insult. In addition, CNTF may promote the activation of surrounding astrocytes in an autocrine/paracrine manner.

#### MANF/CNDF

Mesencephalic Astrocyte-Derived Neurotrophic Factor (MANF) and Cerebral Dopamine Neurotrophic Factor (CDNF) constitute a novel, evolutionary conserved family of NTFs with regenerative capacities in health and disease. Although MANF and CDNF have been originally identified to provide neurotrophic support for dopaminergic neurons, it has become clear that their functions extend beyond those of classical NTFs (161–168). Both NTFs have been associated to numerous tissue-protective and anti-inflammatory functions in models of PD, ischemia and nerve injury (162–164, 168, 169). In addition, a series of recent studies demonstrated that MANF and CDNF are partially retained within the endoplasmic reticulum (ER), where they sense and respond to ER stress by negatively regulating NF-κB dependent inflammatory programs (84, 165, 169–174). In astrocytes, upregulation of both MANF and CDNF has been observed in response to ER stress and experimental stroke, where they alleviate the secretion of pro-inflammatory cytokines IL-1β, TNF-α, and IL-6 (84–86). This is supported by a study using astrocyte-specific overexpression of *Manf*, which resulted in a downregulation of pro-inflammatory cytokines (84). Taken together, this indicates that astrocytic MANF and CDNF function as cell-autonomous safety switch that prevents ER stress induced overactivation and provides neurotrophic support for neurons **(** **Figure 2** **)**. Evidence from a *Drosophila* model of retinal tissue damage further suggests that MANF counteracts the pro-inflammatory functions of VEGF–related factor 1 (Pvf-1) homologue and is required for successful tissue repair (169). This is of particular interest in the context of glial communication, as VEGF secreted by microglia has been demonstrated to induce pro-inflammatory signaling in astrocytes, and the successive MANF secretion by astrocytes may present an anti-inflammatory mechanism that counteracts pathogenic VEGF signaling (48).

### Growth Factors and Neuropoietic Cytokines

#### PDGF Family Members

Platelet-derived growth factors (PDGFs) and their cognate receptors compose a signaling network that consists of five ligand-dimers (PDGF-AA, PDGF-BB, PDGF-AB, PDGF-CC, PDGF-DD) and three receptors (PDGFR-αα, PDGFR-ββ, PDGFR-αβ) (175). While PDGFs have originally been identified as growth factor for smooth muscle cells (176), they are nowadays viewed as potent inducer of oligodendrocyte proliferation and differentiation (177, 178). Interestingly, the PDGF family of cysteine-knot growth factors also includes members of the VEGF subfamily, of which VEGF-B has been shown to induce pro-inflammatory gene expression in astrocytes (48). Similarly, astrocytes can also respond to PDGF-A and PDGF-C by expression of PDGFR-α, which serves as mitogen and inducer of astrocytic branching (179, 180). In addition, a series of studies demonstrated that astrocytes express PDGF-A and PDGF-B monomers, but not PDGF-C or PDGF-D (181–185). In the developing brain, these astrocyte-derived PDGF variants modulate the proliferation and differentiation of oligodendrocyte precursor cells (OPCs) (184, 186) and potentially regulate the proliferation and survival of neurons (187, 188). In the adult CNS, it remains unclear to what extent astrocytes contribute to the PDGF signaling network, as neurons have also been proposed as source of PDGF-A and PDGF-B (189–191). Nevertheless, early work by Silberstein et al. (182) indicates that cultured astrocytes upregulate the expression of PDGFs in response to TNF-α and TGF-β, suggesting a role of PDGF signaling in inflammatory conditions. In this context, two independent studies investigated the therapeutic effects of astrocyte-derived PDGF-A by conditional overexpression in mouse models of chronic and acute CNS demyelination and revealed that elevated expression of PDGF-A by astrocytes significantly increased OPC survival and population density (87, 88) **(** **Figure 2** **)**. While these findings may prove useful to address the progressing demyelination in primary and secondary progressive MS and other degenerative CNS pathologies, important questions remain outstanding. Which programs control the expression of PDGFs in astrocytes? To what extent do astrocyte-derived PDGFs modulate the functions of oligodendrocytes and neurons? And what is their role in the remyelinating brain? Further research into the basic mechanisms of CNS intrinsic signaling of PDGFs is needed to warrant success in their use as future therapeutic target.

#### FGF Family Members

Fibroblast growth factors (FGFs) constitute a family of at least 20 secreted ligands with pleiotropic roles in the developing and mature CNS (192–203). Most FGF receptors (FGFRs) can respond to multiple FGF ligands (e.g. FGFR2 binds FGF1 to FGF10, whereas FGFR3 binds FGF1/2/4/8/9/17/18), creating a complex signaling network where a single FGF can induce distinct cellular responses. This notion is highlighted in a recent article by Duong et al. (196), in which the authors report FGF8 to function as cell fate switch that controls the differentiation of radial glial cells in the SVZ into neurons or astrocytes. Additional studies have demonstrated that FGFs regulate astrocyte morphogenesis, maturation, and function in both health and disease (197–199). For instance, in remyelinating lesions of MS, FGF-1 may act as a promoter of remyelination by an indirect mechanism that involves the induction of CXCL8 and LIF expression in astrocytes (204).

Furthermore, astrocytes have been recognized as important source of FGFs (192). Indeed, reactive astrocytes have been found to upregulate FGF2 expression following CNS insult in multiple species *in vivo* (89, 90) and *in vitro* (205). In particular, a study by Messersmith et al. (90) found significantly increased FGF2 mRNA transcripts and protein levels associated to white matter astrocytes in the initial phase of remyelination, indicating that astrocyte-derived FGF2 may modulate the differentiation of oligodendrocytes (206) **(** **Figure 2** **)**. Other potential effects of astrocyte-derived FGF2 include the attenuation of neuronal death *via* signaling through FGFR3 (207) and autocrine/paracrine regulation of glia reactivity (199). The therapeutic potential of FGF2 is recapitulated in a comprehensive study by Ruffini et al. (208), in which the authors demonstrate that viral delivery of FGF2 to the CNS of mice 1 week after EAE induction significantly ameliorated the clinicopathological outcome, marked by reduced infiltration of peripheral immune cells, and an increase of myelin-forming oligodendrocytes. It is unclear, however, to what extent astrocytes contribute to these beneficial effects of FGF2. Indeed, several reports suggest that FGF2 in general, and astrocyte-derived FGF2 in particular can also inhibit oligodendrocyte repopulation and their remyelinating capacities in multiple models of CNS insult (206, 209–211). Besides FGF2, astrocyte-derived FGF9 has been implicated to play a role during remyelination and CNS inflammation (212). Lindner et al. (212) demonstrated in a series of *in vitro* experiments and post-mortem tissue analyses of MS patients that FGF9, upregulated by astrocytes following CNS insult, inhibits remyelination and induces the expression of inflammatory genes in oligodendrocytes. Overall, the existing data fails to produce a coherent picture on under which conditions FGF family members exhibit beneficial or harmful functions during CNS insult (206, 211) and extensive research is needed to illuminate the effects of astrocyte-derived FGFs. Nevertheless, accumulating evidence strongly suggests that FGFs play an important role in the pathophysiology of MS and (209, 212, 213) and new insights may guide the development of FGF-based therapeutic strategies.

#### HB-EGF

HB-EGF has originally been identified in macrophage-like cells with mitogenic functions for numerous cell types (214). Similar to NGF and other neurotrophins, HB-EGF is synthesized in a pre-mature transmembrane form (pro-HB-EGF) before it is cleaved by numerous metalloproteinases (MMP3, MMP9, ADM9, ADAM10, ADAM12, ADAM17) into its mature, soluble form (215). While the membrane anchored pro-HB-EGF functions as juxtacrine growth factor and receptor for diphtheria toxin in some species, soluble HB-EGF has recently been described to modulate cell migration, differentiation, and inflammatory functions in multiple cell types (216–222). In addition, HB-EGF enhances neurogenesis in models of ischemic injury and promotes the survival of dopaminergic neurons (223, 224) **(** **Figure 2** **)**. Mature HB-EGF signals through EGFR, ErbB4 and a newly defined N-arginine dibasic convertase, but may also be able to induce ErbB2 through heterodimerization (214, 225–227). In astrocytes, upregulation of *HBEGF* mRNA has been observed in response to sphingosine-1-phosphat (S1P)-receptor activation by S1P or S1P receptor modulator fingolimod (92, 93). This may be dependent on combined S1P1R and S1P2R signaling and the activation of the immediate early transcription factors ERG1 and AP1, indicating that astrocyte-derived HB-EGF is part of a rapid response mechanism that counteracts pro-inflammatory astrocyte functions (93). Indeed, it has been suggested that HB-EGF suppresses the nuclear translocation of NF-κB by inhibition of IκB kinase (IKK) mediated inhibitor of κB (IκB) degradation (222). Collectively, astrocyte-derived HB-EGF may not only serve as neurotrophic factor but also dampen pro-inflammatory gene transcription in *Egfr*-expressing microglia and infiltrating immune cells (220).

#### IGF

Insulin-like growth factor 1 (IGF-1) is a polypeptide hormone and functions as primary mediator of growth hormone (GH) dependent growth effects in most peripheral tissues (228). In the brain, IGF-1 regulates the proliferation and differentiation of multiple CNS resident cells and has been implicated in several neurological disorders (229–231). IGF-1 signals through its cognate receptor IGFR-1R, but can also form functional hybrids with the insulin receptor (229). Besides IGF-1, IGF-2 and its receptor IGF-2R share a similar expression pattern in the developing and mature CNS (229). Both IGF/IGFR pairs signal through phosphoinositide 3-kinase (PI3K)–AKT–forkhead box protein O (FOXO) and RAS–mitogen-activated protein kinase (MAPK) pathways to induce downstream expression of growth promoting genes. Interestingly, IGF-1R can furthermore modulate transcription directly by acting as transcriptional regulator in the nucleus (232). Numerous studies indicate roles for IGF-1/IGF-1R signaling in the pathogenesis and progression of neurological disorders and show that their expression is differentially modulated by CNS insult (159, 229, 231, 233, 234). While microglia have been implicated as main source of IGFs under pathological conditions, the neuroprotective potential of astrocyte-derived IGFs has recently gained attention (94–96). For example, conditional overexpression of IGF-1 in astrocytes promoted neuronal survival and reduced hippocampal neurodegeneration in a controlled cortical injury (CCI) model, highlighting the therapeutic efficacy of astrocyte-derived IGFs (96) **(** **Figure 2** **)**. Although the role of endogenous, astrocyte-derived IGFs in the context of neuroinflammation must be further investigated, its broad spectrum of growth promoting effects on CNS-resident cells may provide beneficial for neuronal and non-neuronal regeneration.

#### TGF-β

Transforming growth factor β (TGF-β) belongs to a family of pleiotrophic cytokines with potent regulatory and inflammatory functions in numerous cell types (235–237). In mammals, TGF-β exists in three isoforms (TGF-β1. TGF-β2, TGF-β3), with TGF-β1 being the most prevalent one. The immunoregulatory cytokine elicits its function through binding to TGF-β type I (TGF-βRI) and type II (TGF-βRII) receptors, which induce Smad protein phosphorylation and downstream transcriptional regulation of their target genes (236, 238). Generally, TGF-β has been identified as master regulator of immune tolerance, T cell differentiation and mediator of inflammatory responses in multiple cell types (235, 236, 239, 240). In addition, work by Krieglstein and others suggests that TGF-β also exerts neurotrophic functions through direct or indirect regulation of neuronal development and survival (143, 241–248) **(** **Figure 2** **)**. While members of the TGF-β superfamily are widely expressed among numerous cell types in the CNS, astrocytes have been implicated as key contributor of endogenous TGF-β in the CNS (249). Indeed, astrocyte-derived TGF-β has been linked to anti-inflammatory and neuroprotective functions in models of experimental stroke, Toxoplasma infection, and AD (97, 250, 251). Although the molecular mechanisms underlying the anti-inflammatory and neuroprotective functions of astrocyte-derived TGF-β in the context of neuroinflammation remain to be defined, TGF-β may exert its beneficial role through the suppression of glial NF-κB signaling and the associated pro-inflammatory functions of CNS resident macrophages and microglia (97) **(** **Figure 2** **)**. This is in line with a study defining an IL-10/TGF-β signaling loop between activated astrocytes and microglia that limits CNS inflammation (98). Microglia-derived IL-10, an anti-inflammatory cytokine, redirected astrocyte pathogenic functions and stimulated the production of TGF-β, which in turn reduced microglial activation and the secretion of pro-inflammatory IL-1β (98). Taken together, astrocyte-derived TGF-β may serve as immunosuppressive cytokine during initial inflammation while its neurotrophic functions support axonal regeneration during recovery.

#### LIF

Leukemia inhibitory factor (LIF) is another member of the IL-6 class cytokine family. Analogous to CNTF, LIF signals through LIFRα and gp130 to induce JAK/STAT dependent gene transcription. It was first described as a suppressor of proliferation in a myeloid leukemia cell line, but has since been associated to functions in multiple peripheral organs (252–257). In addition, LIF has been recognized as neuropoetic cytokine, regulating the differentiation and activation of multiple cell types in the CNS (258–262). Under homeostatic conditions, expression of *Lif* remains low in the CNS, but is heavily ramped up in response to various types of insult (99, 100, 263–265). Astrocytes are thought to play an important role in the upregulation of LIF, and have been identified as major source of *Lif* mRNA in the injured brain (100, 263). Consequently, astrocyte-derived LIF may potentiate stem cell renewal in the adult SVZ and increase the regenerative capacities following CNS insult (101, 262) **(** **Figure 2** **)**. Although it is not entirely clear what mechanisms modulate the upregulation of *Lif* expression in astrocytes, S1PR signaling has been shown to be a potent inducer (92, 93). Apart from its beneficial functions on stem cell regeneration and neurogenesis, accumulating evidence shows that LIF plays essential roles during oligodendrocyte maturation and function in the context of autoimmune inflammation and remyelination (102–107). This becomes important both in health and disease. A study by Ishibashi (100) demonstrated that astrocytes secrete LIF in response to ATP stimulation and promote the oligodendrocyte-mediated myelination of axons, defining a mechanism that mediates myelination in an activity-dependent manner. During EAE, increased levels of LIF have been associated to protective functions and increased survival of oligodendrocytes (102, 107) **(** **Figure 2** **)**. In line with this notion, blockage of LIF worsened oligodendrocyte loss while conditional deletion of a LIFR/gp130 suppressor protected against cuprizone-induced demyelination (102, 266). Aside from oligodendrocytes, LIF has been implicated in the regulation of T-cell responses by altering their pathogenic potential. Indeed, several studies show that LIF suppresses pro-inflammatory gene expression in CD4 T-cells and skews their polarization in an anti-inflammatory manner (267–269) **(** **Figure 2** **)**. Collectively, these data suggest that astrocyte-derived LIF may contribute to the resolution of acute tissue inflammation, promote the remyelinating capacities of oligodendrocytes, and induce stem-cell renewal to prevent long-term neurodegeneration.

### Non-Secreted Factors

In addition to neurotrophic factors, neuropoetic cytokines and growth factors, astrocytes secrete a plethora of other protective factors, including cytokines, metabolites, extracellular matrix (ECM) proteins, and metalloproteinases (MMPs) (23, 50, 266, 267, 270). In the healthy brain, tight metabolic coupling between neurons and astrocytes is key to sustain high firing rates and neuronal wellbeing (267). Recently, it has been suggested that the metabolic crosstalk between astrocytes and neurons also plays important roles during neuroinflammation and neurodegeneration (271–273). In this context, it remains to be defined which metabolites with protective functions during homeostasis have similar effects in the inflamed or injured brain. Similarly, astrocyte-derived ECM proteins and MMPs have been associated to numerous protective functions in the healthy brain. However, it has been well documented that astrocyte-derived chondroitin sulfate proteoglycans (GSPGs), which are a key component of the ECM, restrict remyelination, neurite outgrowth and limit functional recovery following CNS injury (274). Among multiple other strategies to overcome CSPG mediated inhibition of neuronal regeneration, MMPs have been proposed to exhibit protective effects in the post-acute phase of CNS injury (275, 276). Indeed, astrocyte-derived MMPs may promote neuronal plasticity in the healthy brain and enhance functional recovery through ECM dependent and independent mechanisms (277, 278). Future research will need to determine which parameters dictate the protective effects of astrocyte-derived ECM components and MMPs, and how they can be harnessed for therapeutic strategies to enhance recovery following CNS insult.

## Therapeutic Outlook and Discussion

Currently, only few effective therapies exist to tackle the vast complexity of neurological disorders and the development of novel strategies is hampered by their limited access to the CNS. Exogenously administered agents require a high permeability through the BBB and a persisting bioavailability to ensure long-lasting therapeutic effects (279). Only a limited number of small molecules has shown beneficial “protective” effects on glial cells following acute CNS insult so far, which is best documented during neuroinflammation (92, 280–286). Thus, there is a dire need for novel strategies that mediate recovery after acute CNS insult and lead to long-term regeneration in chronic inflammatory and degenerative diseases. Based on their strategic location and versatile roles in the pathogenesis and progression of CNS disorders, astrocytes have been proposed as therapeutic targets (230, 231). Generally, most existing approaches targeting astrocytes in the context of neurological disorders are based on gene therapy, cell replacement, or the exogenous administration of compounds that induce neuroprotective functions in astrocytes (287, 288). As discussed above, multiple exogenously administered molecules mimic the protective functions of astrocyte-derived mediators or induce their endogenous production (70, 73, 84, 92, 93, 96, 148, 149, 160). Although these strategies represent promising approaches, several issues will need to be addressed.

First, the multi-faceted functions of astrocyte derived mediators are determined by their differential spatial and temporal expression (18, 289–293). Consequently, exogenous activation of astrocytes at an improper time-point and in the wrong microenvironment might result in harmful, rather than beneficial effects, ultimately worsening clinical outcome. Further research is needed to determine (1) how many functionally distinct astrocyte-subsets exist, (2) which factors induce their differentiation, (3) how the underlying transcriptional programs relate to the differential secretion of astrocyte-derived mediators, and (4) whether these transcriptional subsets also correlate with different secretional and functional astrocyte subpopulations.

Most protective effects mediated by astrocytes are the result of a transient response to environmental cues present in the disease-specific micro-environment. It is conceivable that the diversity and strength of the intercellular crosstalk, specific to a given lesion type, also strongly influences the outcome of specific therapeutic strategies. Indeed, while a transient and highly disease-specific astrocyte response allows for an adapted reaction to the respective insult and prevents extensive overactivation/-suppression, exogenous induction of specific tissue-protective pathways may only provide short-term solutions to long-term problems, and eventually wear off when the local microenvironment changes over the course of the disease.

In these lines, genetic modifications of astrocytes to foster their tissue-protective and anti-inflammatory functions have been proposed (70, 73, 84, 96, 148, 149, 288). These approaches might be particularly useful for the treatment of chronic conditions and allow for the targeted activation of protective subpopulations. In this context, adeno-associated viruses (AAVs) have been proven to be efficient vectors for viral gene delivery. Interestingly, a landmark study by Foust and colleagues (294) demonstrated that AAV serotype 9 successfully bypasses the BBB and predominantly transduces astrocytes in the adult mouse brain (295). Further modifications such as the conditional expression of target genes under the astrocyte-specific GFAP promoter may enhance viral delivery and present a robust delivery system (296, 297). However, AAVs are limited to a relatively small insert size (4.7 kb) and viral delivery may have unpredictable off-target effects. To overcome this complication, cell replacement strategies (using genetically modified or unmodified cells) may present a useful alternative to harness the anti-inflammatory and tissue-protective functions of astrocytes. Several transplantation trials of human fetal mesencephalic stem cells into striatal regions of PD patients have demonstrated successful functional integration and long-term benefits (298–302). This is different for astrocytes, as they require differentiation into their mature form before being grafted. Although we have little information about whether human astrocytes can be generated from embryonic stem cells (ESCs) or NSCs, several studies report successful and stable differentiation of human ESCs into dopaminergic neurons, and transplantation of glial-restricted pluripotent stem cells in mouse models of ALS suggest that this might represent a feasible approach (303–307). One significant advantage over existing cell replacement therapies using neurons is that one astrocyte has the capacity to induce differentiation and survival in numerous neurons (through the secretion of soluble factors), thus making it an efficient approach to tackle neurodegeneration. Indeed, two ongoing Phase I/IIa trials (NCT03482050, with GDNF overexpression NCT02943850) currently examine the therapeutic potential of grafted human stem cell derived astrocytes for the treatment of ALS.

Overall, numerous studies presented in this review suggest that exogenous application or the genetic overexpression of astrocyte derived factors limit inflammation and aid central nervous regeneration. These findings need to be strengthened and extensively recapitulated in other clinically relevant model species and CNS injuries before taking the next step towards clinical application. Such models will allow to find common mechanisms underlying tissue-protective functions of astrocytes and assess their translatability in a defined setting. Furthermore, it will become essential to investigate the combinatorial effects of astrocyte-derived factors as multiple studies have demonstrated synergistic effects and cross-regulatory mechanisms between several of the discussed mediators (308–315). Lastly, we are just beginning to grasp the versatile roles glial cells play in the diseased CNS and the extensive characterization of astrocytic subsets beyond a dualistic concept will be inevitable to understand their roles in the context of CNS inflammation. Novel high-throughput technologies will pave the way for a better understanding of what signals drive the secretion of protective factors by astrocytes, to what extent these transcriptional profiles are influenced by intercellular communication, and how we can harness the protective potential of reactive astrocytes in a clinical setting.

## Author Contributions

Both ML and VR researched data and reviewed and edited the manuscript. ML wrote the manuscript.

## Funding

This work was supported by grants RO4866/2-1 and RO4866/4-1 from the German Research foundation (DFG) and an ERC Starting Grant (851693 – HICI) by the European Research Council.

## Conflict of Interest

The authors declare that the research was conducted in the absence of any commercial or financial relationships that could be construed as a potential conflict of interest.
